# Tetraimido Sulfuric Acid H_2_S(N*t*Bu)_4_—Valence Isoelectronic to H_2_SO_4_

**DOI:** 10.1002/anie.202014426

**Published:** 2021-01-28

**Authors:** Jochen Jung, Annika Münch, Regine Herbst‐Irmer, Dietmar Stalke

**Affiliations:** ^1^ Georg-August Universität Göttingen Institut für Anorganische Chemie Tammannstrasse 4 37077 Göttingen Germany

**Keywords:** charge density, ligand design, metal coordination, structure determination, sulfur nitrogen ligands

## Abstract

The valence isoelectronic imido analog H_2_S(NtBu)_4_ (**1**) of sulfuric acid H_2_SO_4_ was synthesized, isolated, and characterized by NMR spectroscopy and high‐resolution X‐ray charge density analysis. The latter reveals strongly polarized S^δ+^−N^δ−^ bonds with virtually no double bond character. The easy‐to‐polarize S−N bonds are an advantageous and versatile feature of sulfur nitrogen ligands, which enables them to adapt to different electron requirements of various metal cations.

## Graphical abstract

H_2_S(N*t*Bu)_4_ was synthesized, isolated and characterized by NMR spectroscopy and high‐resolution X‐ray charge density analysis. The topological parameters *ρ*(**r**), ∇^2^ *ρ*(**r**), and *ϵ* characterize the two different amido S−N(H)*t*Bu and imido S−N*t*Bu bonds as strongly polarized single bonds. The entire SN_4_ unit can easily adapt to different electronic requirements.

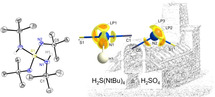

Isovalent electronic replacement of the oxygen atoms in the classic SO_*n*_^*m*−^ polyoxo sulfur anions by NR imido groups gives molecular soluble polyimido sulfur species S(NR)_*n*_^*m*−^, *n*=2, 3, 4 and *m*=0, 2 (Scheme 1[^1^, ^2^, ^3^, ^4^, ^5^]). They are multifunctional ligands in metal coordination.[^6^, ^7^] Unequivocally, the sulfur nitrogen bond is one of the most versatile in coordination chemistry and materials science. This results from the wide range of oxidation states of sulfur (−II to +VI), permitting easy metal‐to‐ligand charge transfer, the large radius, enabling high coordination numbers, and the considerable covalent but polar nature of S−N bonds. The synthesis of polymeric (SN)_*x*_ ^[8]^ and the discovery of its high‐temperature superconducting properties^[9]^ certainly were some of the milestones in SN chemistry, recently stimulated by the synthesis of the nitrogen‐poor sulfur nitride oxide N{S(O)_2_O(O)_2_S}_3_N.^[10]^ In addition, sulfur‐centered ligands turned out to be advantageous donors^[11]^ in single‐molecule magnets (SMM) and charge density investigations helped to experimentally assign the electronic configuration of the metal center in SMMs.^[12]^ In the past we have been already successful employing those ligands to d block SMMs.^[13]^ This fuelled the idea to synthesize and isolate tetraimido sulfuric acid H_2_S(N*t*Bu)_4_ as a major cornerstone building block in SN chemistry.

**Scheme 1:**
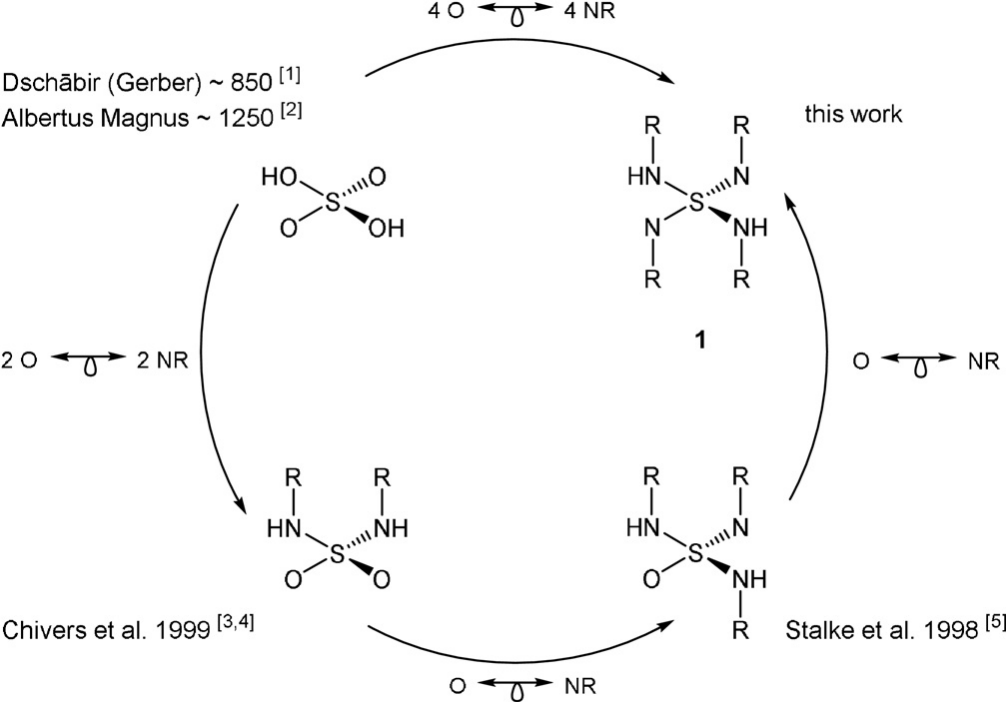
From sulfuric acid to tetraimido sulfuric acid by isovalent electronic replacement of O by NR.

Gentle protonation of the lithiated tetraimido sulfate [(thf)_4_Li_2_(N*t*Bu)_4_S]^[5]^ with *t*BuNH_3_Cl in thf at room temperature^[14]^ gives the long sought text‐book compound H_2_S(N*t*Bu)_4_ (**1**, Scheme 1), a valence isoelectronic imido analog to sulfuric acid H_2_SO_4_,^[15]^ the most important bulk product of chemical industry.^[16]^ This compound turned out to be unstable in solution as well as in the solid state, even at low temperatures. It is only storable at temperatures below −35 °C without significant decomposition for several months. Elevated temperatures generate increasing impurities of S(N*t*Bu)_3_ (**2**), the analog to SO_3_ and starting product of H_2_SO_4_.^[17]^ Similar solubilities render fractional crystallization impossible and attempted high‐vacuum sublimation only results in isolation of S(N*t*Bu)_3_. The other major product is *tert*‐butylamine (see Scheme S1 and Figure S17). The ratio of **1** and **2** can be determined via ^1^H NMR spectroscopy and integration of the resulting signals, sufficiently different in their chemical shift of 1.28 ppm and 1.51 ppm for **1** and 1.44 ppm for **2** (Figure S11). The signal of the two equivalent nitrogen‐bound protons of **1** can be found at 3.06 ppm and the ^15^N‐^1^H HSQC (Figure S15) and ^15^N‐^1^H HMBC NMR spectra (Figure S16) show them to have the same nitrogen environment as the *t*Bu groups resonating at 1.28 ppm. The N*t*Bu groups at 1.51 ppm do not show any NH interference. Additionally, the ^15^N NMR chemical shifts of the nitrogen atoms in **1** (−253.2 ppm for N and −257.4 ppm for NH) are different to those of **2**. Despite all the preparative obstacles we finally succeeded in the crystallization, isolation, and high‐resolution structural characterization of pure **1**. It crystallizes in the space group *C*2/*c* with no indication of any N(H)/N hydrogen atom disorder (Figure 1), therefore it would be more appropriate to stress the fixed hydrogen positions and the nature of the two different substituents in a formula like [(*t*BuN)_2_S(HN*t*Bu)_2_]. The asymmetric unit contains only half of the molecule with a twofold axis running through the sulfur atom and the N‐S‐N and the (H)N‐S‐N(H) bisections, respectively. Hence the sulfur atom adopts a distorted tetrahedral geometry, created by two N*t*Bu imido and two (H)N*t*Bu amido groups each.

**Figure 1:**
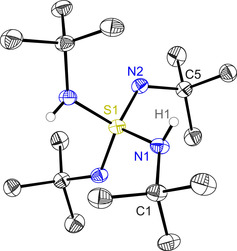
Crystal structure of H_2_S(N*t*Bu)_4_ (**1**). Anisotropic displacement parameters are depicted at the 50 % probability level. Carbon‐bound hydrogen atoms are omitted for clarity. Selected bond lengths [Å] and angles [°]: S1–N1 1.6482(3), S1–N2 1.5273(3), N1–C1 1.4902(3), N2–C5 1.4776(3); N1‐S1‐N1a 113.00(2), N1‐S1‐N2 100.750(13), N2‐S1‐N2a 127.83(2), N1‐S1‐N2a 107.392(13).

The largest bond angle is found between two imido groups (N2‐S1‐N2A 127.83(2)°) and the smallest is enclosed by the amido/imido groups (N1‐S1‐N2 100.750(13)°). The averted orientation of the N1(H) hydrogen atom and the N2 lone pairs prevents any intra‐ as well as intermolecular hydrogen bonding. The imido bond length of S1−N2 of 1.5273(3) Å and the amido bond length of S1−N1 of 1.6482(3) Å correspond very well to already determined distances in S(N*t*Bu)_3_ (**2**),^[19]^ S(N*t*Bu)_2_ (**3**),^[20]^ and CH_2_{S(N*t*Bu)_2_(NH*t*Bu)}_2_ (**4**)^[21]^ in the gas phase[^22^, ^23^] and on the basis of high‐resolution X‐ray data in the solid state (Table 1). To elucidate the charge density distribution in **1**, especially the bonding situation of the different S−N bonds, vital for the single molecule magnet (SMM) behavior, we performed a multipole refinement with the Hansen and Coppens formalism,^[24]^ followed by Quantum Theory of Atoms in Molecules (QTAIM)‐based topological analysis.^[25]^

**Table 1:** Topological parameters of selected bonds in **1**–**4**.^[a]^

Compound	Bond	*ρ*(*r*_BCP_) [e Å^−3^]	∇^2^ *ρ*(*r*_BCP_) [e Å^−5^]	*d*_BP_ [Å]	*d*_BCP‐A1_ [Å]	*d*_BCP‐A2_ [Å]	*ϵ*	Ref.
H_2_S(N*t*Bu)_4_ (**1**)	S1−N1(H)	1.808(6)	−14.8(3)	1.64860(15)	0.805(3)	0.843(2)	0.43	this work
	S1−N2	2.173(4)	−21.2(3)	1.5429(2)	0.740(2)	0.789(3)	0.32	
S(N*t*Bu)_3_ (**2**)	S1−N1	2.27(3)	−10.56(8)	1.513	0.738	0.775	0.22	[19]
S(N*t*Bu)_2_ (**3**)	S1−N1	1.93(3)	−9.44(8)	1.546	0.681	0.865	0.07	[20]
	S1−N2	2.24(3)	−9.38(7)	1.531	0.788	0.743	0.07	
CH_2_{S(N*t*Bu)_2_(NH*t*Bu)}_2_ (**4**)	S1−N1(H)	1.89(2)	−13.41(7)	1.650	0.780	0.870	0.11	[21]
	S1−N2	2.31(3)	−16.60(9)	1.530	0.718	0.812	0.10	
	S1−N3	2.37(3)	−16.44(9)	1.520	0.718	0.802	0.06

[a] *d*_BP_: Bond path length, *d*_BCP‐A1/2_: distance of BCP to atom A1/2, *ρ*(r_BCP_): electron density at BCP, ∇^2^ *ρ*(r_BCP_): Laplacian values at BCP, *ϵ*: ellipticity at BCP. Estimated standard deviations were determined by standard deviation of 20 cross‐validation sets.^[18]^

Important quantities to characterize the bonding are the electron density *ρ*(**r**) (ED), Laplacian ∇^2^ *ρ*(**r**) (second derivative of *ρ*(**r**)), and ellipticity *ϵ* at the bond critical point (BCP). The nature of the S−N bond is of particular interest, especially with respect to a single and double bond character as well as the polarity. Furthermore, the ED distribution around the central sulfur atom is of great interest, because it is a key component of ligands leading to SMMs. The ED at the BCP at the bond path is analyzed first. A BCP is defined as the local minimum in electron density from which gradient paths proceed with increasing slope. Within QTAIM such a path is called a bond path (BP), which is a sufficient and necessary condition for a chemical bond. It has to be emphasized, however, that this interaction neither has to be attractive^[26]^ nor has it to be a classical two‐center two‐electron bond.^[27]^

Selected S−N bond properties of **1**–**4** are summarized in Table 1. In general, it was found for all compounds that the position of the BCP (*d*_BCP_) is shifted towards the electropositive sulfur atom. This is a distinct indication for an electronically depleted sulfur atom and a polar bond. In **1** the BCP of the amido S1−N1(H) bond is shifted slightly less in the direction of the sulfur atom, so that a lower polarization is observed here than with the imido S1−N2 bond. This observation agrees well with the values of the S1−N1(H) bond from compound **4**. Nevertheless, based on the *ρ*(**r**) and ∇^2^ *ρ*(**r**) at the BCP, the bonds can be characterized as shared interactions. Both values support the covalent bond character of the S−N, already established for the sulfur triimide **2** and the sulfur diimide **3**. However, they have to be interpreted as polar single bonds with virtually no double bond character. The electron densities at the S−N BCPs in **1** are not identical and slightly higher by 0.37 [e Å^−3^] at the imido bond. Figure 2 shows the molecular graph of the two crystallographically independent bonds. Both bond paths are not straight and especially S1−N2 displays a pronounced curvature, symptomatic for polar bonds.^[28]^ Furthermore, it is worth mentioning that the ellipticity of both S−N bonds is unusually high (0.43 and 0.32, respectively), even compared to **2** and **3**. In a non‐polar C−C bond this value would usually be associated with the double bond character due to the π density accumulated above and underneath the cylindrical σ bond. Things are different here: the lone‐pair density at each nitrogen atom wants to balance the high positive charge of the sulfur atom and leaps into the bonding region, causing the deformation of the otherwise cylindrical σ density (Figure 3). Interestingly, the sum of all four S−N bond distances in **1** (6.351 Å) falls in the range of all other [S(N*t*Bu)_4_]^2−^ tetraimido anions, regardless to the coordinated metal (from 6.343 Å for Cu^II^ to 6.395 Å for Li/Zn).^[29]^ The SN_4_ unit obviously responds flexibly to different electronic requirements induced by either different metal cations in terms of the sulfur atom being shifted relative to an otherwise almost fixed tetrahedral N_4_ environment.^[30]^ This experimentally emphasizes the high polarization of the S−N bonding rather than valence expansion and d orbital participation in S−N multiple bonding.^[20]^ The analysis of the Laplacian in the non‐bonding regions of the nitrogen atoms corroborates the argument that S1−N2 is not a double bond. One valence shell charge concentration (VSCC) at the apical non‐bonding region of the amide nitrogen N1 could be found (∇^2^ *ρ*(**r**)_max_(LP1)=−57.4 e Å^−5^), as well as two VSCCs at the imido nitrogen atom N2 (∇^2^ *ρ*(**r**)_max_(LP2)=−57.3 e Å^−5^, ∇^2^ *ρ*(**r**)_max_ (LP3)=−47.9 e Å^−5^) (Figure 3). VSCCs in non‐bonding regions are indicative for lone pairs (LPs). The two VSCCs at the imido nitrogen atom N2 complete an even more distorted tetrahedron, equally leaning over to the electropositive sulfur atom. The geometry at N1 including the LP describes a distorted tetrahedron geometry with LP1 in the apical position. The LP1 and LP2 isosurfaces nicely depict the lambent density towards the sulfur atom.

**Figure 2:**
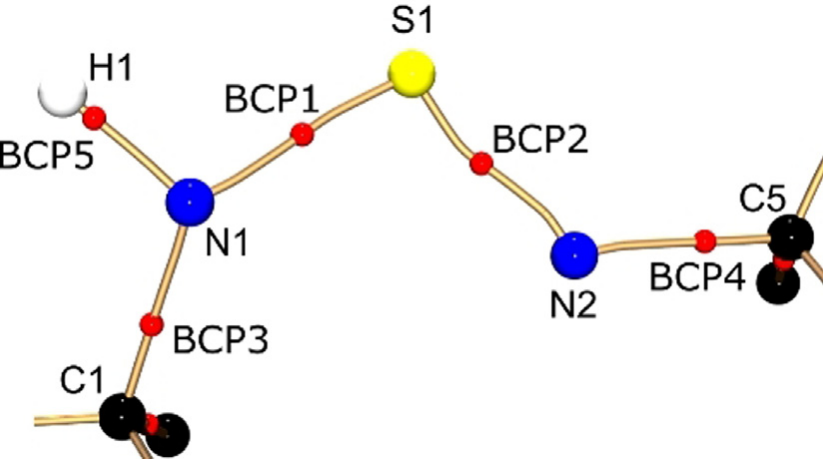
Molecular graph of the central part of **1**. Bond paths are colored in bronze, BCPs depicted by red spheres.

**Figure 3:**
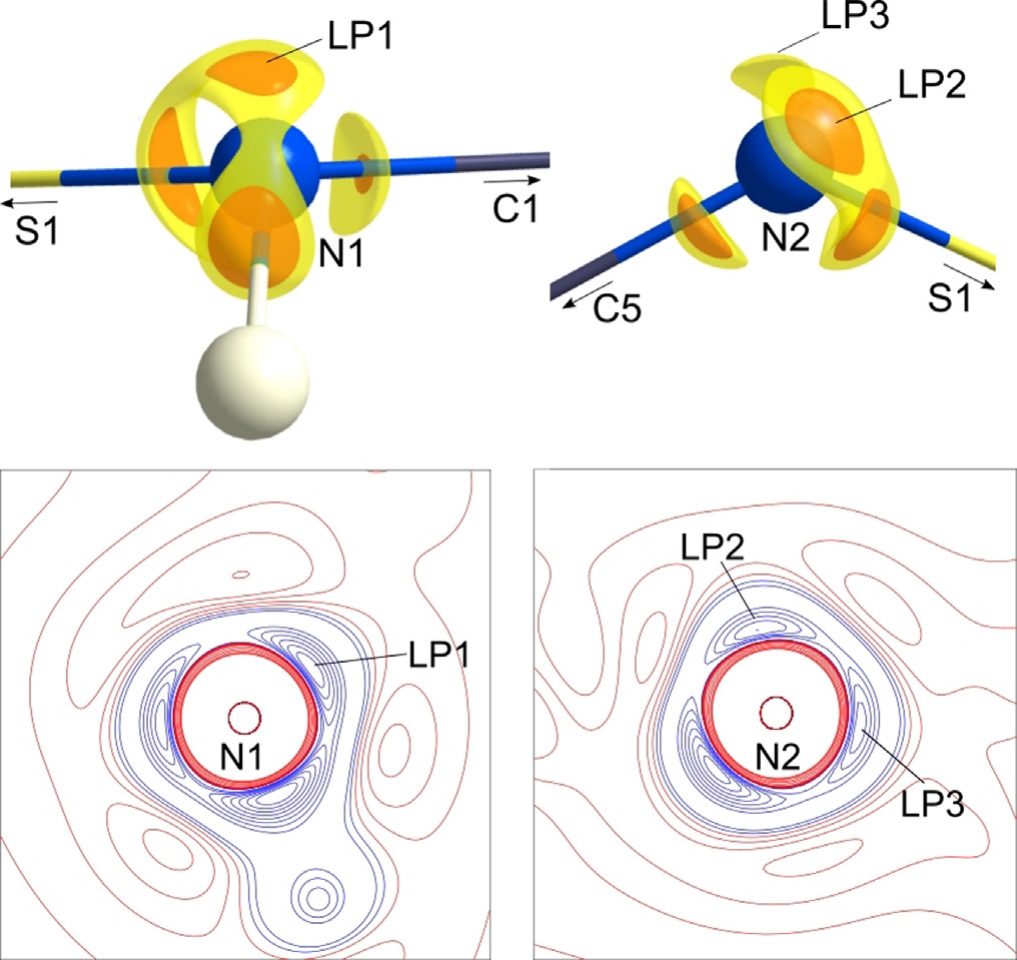
∇^2^ *ρ*(**r**) at an isolevel of −52 e Å^−5^ (orange) and −35 e Å^−5^ (yellow) at N1 and N2 in **1** and contour plots of charge concentrations in the H1‐N1‐LP1 and LP2‐N2‐LP3 plane. Contours are drawn at ±(1, 3, 20, 25, 30, 35, 40, 45, 50, 55, 70, 100, 115, 135) e Å^−5^, blue contours show negative values, red values show positive values.

In conclusion, for the first time we were successful in the synthesis and isolation of H_2_S(N*t*Bu)_4_ (**1**), a valence isoelectronic imido analog to sulfuric acid H_2_SO_4_. Furthermore, we were able to characterize this text‐book molecule by NMR spectroscopy and high‐resolution X‐ray charge density analysis. A detailed analysis of the experimental electron density with the topological parameters *ρ*(**r**), ∇^2^ *ρ*(**r**), and *ϵ* characterizes the two different S−N bonds. It could be established that the amido S−N(H) and the imido S−N bonds are strongly polarized single bonds. The analysis of the Laplacian reveals a non‐symmetrical distribution of the VSCCs around the nitrogen atoms, cantilevered towards the sulfur atom, explaining the high bond ellipticities. The polarizability offers the advantage that the entire SN_4_ unit can adapt to different electronic requirements of various coordinated metals.^[31]^ This kind of flexibility towards electronic interactions between ligand and metal indicates a promising system for the formation of single molecule magnets. The dual N(H) functionality opens a wide avenue to soluble (hetero)bimetallic complexes.

## Experimental Section

**Synthesis of H_2_S(N*t*Bu)_4_ (1)**: A mixture of [(thf)_4_Li_2_(N*t*Bu)_4_S] (100.0 mg, 0.1616 mmol) and *t*BuNH_3_Cl (39.0 mg, 0.356 mmol) was dissolved in tetrahydrofuran (2 mL) at ambient temperature. After stirring for 2 min the solvent was removed under reduced pressure. The residue was dissolved in *n*‐pentane (1 mL) and the solution was filtered to remove LiCl. The filtrate was concentrated again, washed with MeCN (0.2 mL), and stored at −35 °C. Crystallization started within minutes yielding colorless crystals suitable for X‐ray analysis. The product was isolated and dried under reduced pressure. Yield: 44.1 mg (69 %); ^1^H‐NMR (500.13 MHz, 283 K, C_6_D_6_): *δ* [ppm]=1.28 (s, 18 H, 2 HNC(C*H*_3_)_3_), 1.51 (s, 18 H, 2 NC(C*H*_3_)_3_), 3.06 (s, 2 H, 2 *H*NC(CH_3_)_3_); ^13^C{^1^H}‐NMR (125.76 MHz, 283 K, C_6_D_6_): *δ* [ppm]=30.6 (s, HNC(*C*H_3_)_3_), 32.96 (s, NC(*C*H_3_)_3_), 52.95 (s, N*C*(CH_3_)_3_), 53.81 (s, HN*C*(CH_3_)_3_); ^15^N‐NMR (50.70 MHz, 283 K, C_6_D_6_): *δ* [ppm]=−257.41 (*N*C(CH_3_)_3_), −253.19 (H*N*C(CH_3_)_3_).

All experiments were performed under inert conditions in N_2_ or Ar atmosphere using Schlenk techniques or in an Ar glovebox. Solvents were dried over sodium or potassium, distilled prior to use and stored over molecular sieves (3 Å). Starting materials were purchased commercially and used without further purification. [(thf)_4_Li_2_(N*t*Bu)_4_S] was synthesized according to a known literature procedure.^[5]^ NMR spectra were measured on a Bruker Avance III HD 500 and referenced to deuterated solvent signal. The single crystal for high‐resolution X‐ray charge density analysis was selected under cooling using the X‐Temp2 device.^[32]^ The dataset was collected at a Bruker SRA TXS‐Mo rotating anode with mirror optics and an APEX II detector with a D8 goniometer. The data were integrated with SAINTv8.38A.^[33]^ A multi‐scan absorption correction was applied using SADABS^[34]^ and a 3 *λ* correction was performed.^[35]^ The structure was solved by SHELXT^[36]^ and refined on *F* ^2^ using SHELXL^[37]^ in the graphical user interface ShelXle.^[38]^ Afterwards a multipole refinement and the topological analysis were performed in the XD2006 program package.^[39]^ Crystal data for **1** at 100(2) K, *Mr*=318.56 g mol^−1^, 0.25×0.26×0.35 mm, monoclinic, *C*2/*c*, *a=*17.133(3) Å, *b=*8.612(2) Å, *c=*15.278(2) Å, *β*=117.14(2)°, *V=*2006.1(7) Å^3^, *Z*=4, *μ*(Mo Kα)=0.163 mm^−1^, 2*θ*_max_=90.74°, R1(*F* ^2^)=0.0248, wR1(*F* ^2^)=0.0273, res. density peaks: 0.187 to −0.123 e Å^−3^. The Crystallographic Information Files (CIFs) can be obtained free of charge from the joint Cambridge Crystallographic Data Centre and Fachinformationszentrum Karlsruhe Access Structures service using the deposition number 2023910.

## Conflict of interest

The authors declare no conflict of interest.

## Supporting information

As a service to our authors and readers, this journal provides supporting information supplied by the authors. Such materials are peer reviewed and may be re‐organized for online delivery, but are not copy‐edited or typeset. Technical support issues arising from supporting information (other than missing files) should be addressed to the authors.

Supplementary
