# Supplementary material for: Tetraimido Sulfuric Acid H2S(NtBu)4—Valence Isoelectronic to H2SO4
Source: Angew Chem Int Ed Engl. 2021 Jan 28;60(11):5679–82. doi: 10.1002/anie.202014426 (PMC7986191; doi:10.1002/anie.202014426)
Supplement: Supplementary file 1 — Supplementary [file ANIE-60-5679-s001.pdf]

Angewandte Chemie
Eine Zeitschrift der Gesellschaft Deutscher Chemiker

Supporting Information

Tetraimido Sulfuric Acid $H_2S(NtBu)_4$—Valence Isoelectronic to H_2SO_4

*Jochen Jung[+], Annika Münch[+], Regine Herbst-Irmer, and Dietmar Stalke**

anie_202014426_sm_miscellaneous_information.pdf

Supporting Information

Table of Contents

Table of Contents 1
S1. High resolution XRD-Analysis of 1 3
S1-1. Data collection 3
S1-2. Multipole Refinement 3
S1-3. Rcross Validation[12] 5
S1-4. Refinement of anharmonic motion[13] 7
S1-5. Data quality [15] 9
S1-6. Topological Analysis 10
S2. NMR spectra 12
S3. Synthesis and decomposition of 1 15

J. Jung, A. Münch, Dr. R. Herbst-Irmer, Prof. Dr. D. Stalke
Universität Göttingen
Institut für Anorganische Chemie
Tammannstraße 4, 37077 Göttingen, Germany
E-mail: dstalke@chemie.uni-goettingen.de

Supporting information for this article is given via a link at the end of the document.

Table S1. Crystallographic details at 100K.

compound	**1**
Empirical formula	C_{16} H_{38} N_4 S
Mol. w. (g mol^{-1})	318.56
CCDC no.	2023910
Temperature (K)	100(2)
Wavelength (Å)	0.71073
Crystal system	monoclinic
Space group	C2/c
a (Å)	17.133(3)
b (Å)	8.612(2)
c (Å)	15.278(2)
β (°)	117.14(2)
V (Å^3)	2006.1(7)
Z	4
Max. res. (Å)	0.50
Refl. measured	211506
Refl. unique	8338
R_{int}	0.0389
**IAM refinement**	
Data / restraints / parameters	8338 / 1 / 106
R1 (I > 2σ(I))	0.0284
wR2 (all data)	0.0988
GOF	1.060
Largest diff. peak and hole (eÅ^{-3})	0.460 / -0.385
**Multipole refinement**	
R1(F^2)	0.0248
wR1(F^2) (all refl.)	0.0273
GOF	1.88
Largest diff. peak and hole (eÅ^{-3})	0.187 / -0.123

S1. High resolution XRD-Analysis of 1

Figure S1. Crystal structure of **1**. Anisotropic displacement parameters are depicted on a probability level of 50%. All ^{t}Bu- hydrogen atoms are omitted for clarity.

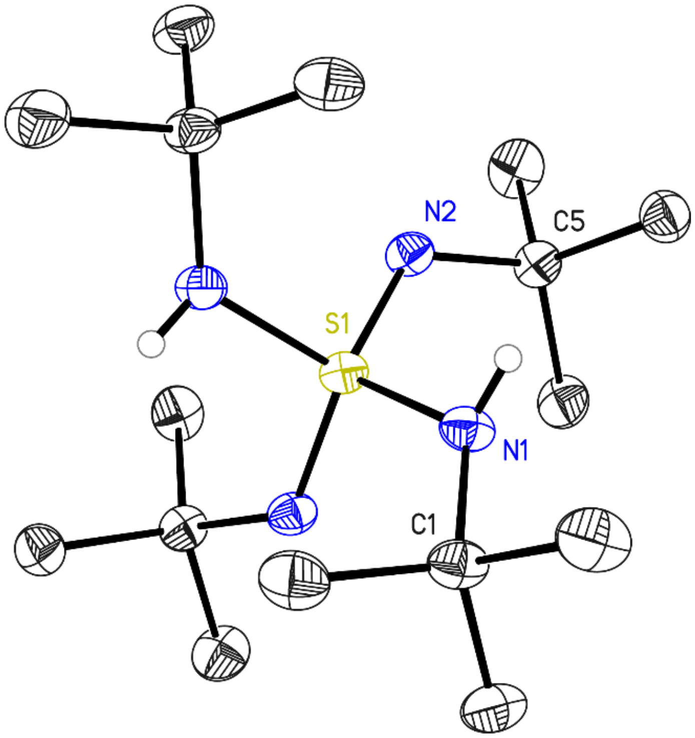

S1-1. Data collection

High resolution single X-ray data were collected on a Bruker SRA TXS Mo rotating anode with APEX II detector at 100 K. The single crystal was mounted from inert oil at low temperature and under nitrogen atmosphere using the X-Temp 2 device[1]. The data were collected with 15 low-angle runs at exposure times up to 15 s (d_{min} = 0.9 Å ($2\theta_{max}$ = -24.1°)), 11 medium-angle runs at exposure time up to 30 s (d_{min} = 0.7 Å ($2\theta_{max}$ = -44.6°)) and 26 high-angle runs at exposure times up to 120 s (d_{min} = 0.49 Å ($2\theta_{max}$ = -90.4°)).
The integration was performed with Saint v8.38A.[2] For the integration a fixed box size (x=0.3°, y=0.3°, z=0.4°) was used. The reflection 1 0 0 was omitted, because it is hidden on some frames by the shadow of the beam stop and is overexposed on other frames. Afterwards, the data set was corrected for absorption, scaled and merged with Sadabs-2016/2 with error model 1.[3] A 3 λ correction with a correction factor of 0.0015 was performed.[4] Systematic absences and reflections with negative intensities were omitted. The structures was solved by SHELXT[5] and a starting model was refined on F^2 using SHELXL[6] in the graphical user interface ShelXle.[7] Afterwards a multipole refinement and the topological analysis were performed in the XD2006 programme package.[8]

S1-2. Multipole Refinement

The multipole refinement (aspherical-atom formalism) using the nucleus-centred multipole model of Hansen & Coppens was carried out on F^2 with the full-matrix-least-squares refinement program XDLSM implemented in the XD2016/XD2006.542 program[8] using experimental weights. The core and the spherical valence densities were composed of relativistic Dirac-Fock wave functions reported by Su, Coppens and Macchi[9] (SCM bank file). The radial fit of these functions was optimized by the expansion-contraction parameters κ and κ'. The expansions over the spherical harmonics were truncated at the hexadecapolar level for all non-hydrogen atoms and all multipoles (nl = 1 to 4) of each atom shared the same κ'-set (KEEP KAPPA constraint). For the hydrogen atoms bond directed dipoles were refined, while expansion-contraction parameters were kept fixed at κ=1.1 and κ'=1.18. Moreover, the *U*iso values of the hydrogen atoms were constrained to 1.5 *U*eq of their pivot atoms. After each step the distances of the hydrogen atoms were reset to their

distances from neutron diffraction experiments.[10] Some of the atoms in the structure showed effects of anharmonic motion.[11] These atoms (S1, C2, C4, C6) were refined using the Gram-Charlier coefficients up to 3rd order.

For stabilizing the refinement, a maximum amount of chemical constraints and symmetry restrictions for the multipolar functions were applied for the first refinement steps. In the final refinement stages, some constraints for similar atoms were dismissed (Tab.S1-3). The density parameters were introduced in the refinement routines in a stepwise manner but in the final cycles all refineable parameters (except κ') were refined together until convergence was reached.

Table S2. Local coordinate systems, site symmetry and chemical constraints of the start model

ATOM	ATOM0	AX1	ATOM1	ATOM2	AX2	SITESYM	CHEMCON
S(1)	DUM0	Z	S(1)	N(1)	X	mm2	
N(1)	S(1)	Z	N(1)	C(1)	Y	Nosymm	
N(2)	S(1)	Z	N(2)	N(1)	X	mx	
C(1)	N(1)	Z	C(1)	C(3)	Y	3zmx	
C(2)	C(1)	Z	C(2)	H(2C)	Y	3zmx	
C(3)	C(1)	Z	C(3)	H(3A)	Y	3zmx	C(2)
C(4)	C(1)	Z	C(4)	H(4C)	Y	3zmx	C(2)
C(5)	N(2)	Z	C(5)	C(8)	Y	3zmx	C(1)
C(6)	C(5)	Z	C(6)	H(6C)	Y	3zmx	C(2)
C(7)	C(5)	Z	C(7)	H(7C)	Y	3zmx	C(2)
C(8)	C(5)	Z	C(8)	H(8B)	Y	3zmx	C(2)
H(1)	N(1)	Z	H(1)	C(1)	Y	cy	
H(2A)	C(2)	Z	H(2A)	H(2C)	Y	cy	
H(2B)	C(2)	Z	H(2B)	H(2C)	Y	cy	H(2A)
H(2C)	C(2)	Z	H(2C)	H(2B)	Y	cy	H(2A)
H(3A)	C(3)	Z	H(3A)	H(3B)	Y	cy	H(2A)
H(3B)	C(3)	Z	H(3B)	H(3A)	Y	cy	H(2A)
H(3C)	C(3)	Z	H(3C)	H(3B)	Y	cy	H(2A)
H(4A)	C(4)	Z	H(4A)	H(4C)	Y	cy	H(2A)
H(4B)	C(4)	Z	H(4B)	H(4C)	Y	cy	H(2A)
H(4C)	C(4)	Z	H(4C)	H(4A)	Y	cy	H(2A)
H(6A)	C(6)	Z	H(6A)	H(6B)	Y	cy	H(2A)
H(6B)	C(6)	Z	H(6B)	H(6A)	Y	cy	H(2A)
H(6C)	C(6)	Z	H(6C)	H(6A)	Y	cy	H(2A)
H(7A)	C(7)	Z	H(7A)	H(7C)	Y	cy	H(2A)
H(7B)	C(7)	Z	H(7B)	H(7C)	Y	cy	H(2A)
H(7C)	C(7)	Z	H(7C)	H(7B)	Y	cy	H(2A)
H(8A)	C(8)	Z	H(8A)	H(8C)	Y	cy	H(2A)
H(8B)	C(8)	Z	H(8B)	H(8A)	Y	cy	H(2A)
H(8C)	C(8)	Z	H(8C)	H(8A)	Y	cy	H(2A)

Table S3. .Fractional coordinates of the dummy.

DUM0	0.5	0.829473	0.75

S1-3.Rcross Validation[12]

Abbreviations: **M**: monopoles; **HM**: monopole for hydrogen atoms; **HD**: bond directed dipole for hydrogen atoms; **D**: dipoles; **Q**: quadrupoles; **O**: octupoles; **H**: hexadecapoles, **U2**: Uij, **k**: kappa, **U3**(atom name): Gram Charlier 3rd order; **nosymm**: no local symmetry constraints, **nocon**: no chemical constraints; **tBu**: carbon atoms in ᵗbutyl group; **d/p**: data to parameter ratio; **GOF**. Goodness of Fit; **low/MP**: low-order-data to multipole parameter ratio. The scale factor is refined in every step but only mentioned in the first. The new added parameter is marked in red while the last refinement step with model improvement and no overfitting is marked in green.

Table S4. Refinement steps 1-20.

Step	Parameter	#p	#d	D2p	R(1F^2)	Low/MP	GOF
1	Scale factor	1	6499	6499	0.0632	0	7.351
2	**D Q O (all) HD**	40	6499	162.5	0.0366	26.1	4.022
3	**M** D Q O **HM** HD	46	6499	141.3	0.0357	22.1	3.804
4	**U2** M D Q O HM,HD	110	6499	59.1	0.0354	22.1	3.641
5	**XYZ** U2 M D Q O HMHD	141	6499	46.1	0.033	22.1	3.194
6	XYZ U2 M D Q O HM HD **k**	146	6499	44.5	0.0328	19.9	3.094
7	**HXYZ**	58	1017	17.5	0.0311	0	4.802
8	XYZ U2 M D Q O HM HD k	146	6499	44.5	0.0305	19.9	2.617
9	XYZ U2 M D Q O **H** HM HD k	167	6499	38.9	0.0275	14.1	2.337
10	**k'**	6	6499	1083.2	0.0271	0	2.276
11	XYZ U2 M D Q O H HM HD k	167	6499	38.9	0.0269	14.1	2.275
12	XYZ U2 M D Q O H HM HD k sigobs[0]	167	7844	47	0.0269	14.6	2.092
13	XYZ U2 U3 (S1,C2,C4,C6) M D Q O H HM HD k	201	7844	39	0.026	14.6	1.976
14	XYZ U2 U3 (S1,C2,C4,C6) M D Q O H HM HD k nosymm @N(2)	211	7844	37.2	0.0256	12.8	1.952
15	XYZ U2 U3 (S1,C2,C4,C6) M D Q O H HM HD k nosymm @N(2) 2z@S1	215	7844	36.5	0.0253	12.2	1.924
16	XYZ U2 U3 (S1,C2,C4,C6) M D Q O H HM HD k nosymm @N(2) 2z@S1 nocon C1/C5	222	7844	35.3	0.0251	12.2	1.911
17	XYZ U2 U3 (S1,C2,C4,C6) M D Q O H HM HD k nosymm @N(2) 2z@S1 nocon C1/C5 3z @C1/C5	224	7844	35	0.0251	11.9	1.911
18	XYZ U2 U3 U3 (S1,C2,C4,C6) M D Q O H HM HD k nosymm @N(2) 2z@S1 nocon C1/C5 3z @C1/C5 C2	228	7844	34.4	0.0251	11.7	1.905
19	XYZ U2 U3 (S1,C2,C4,C6) M D Q O H HM HD k nosymm @N(2) 2z@S1 nocon C1/C5 mx @C2 nosymm @C2	244	7844	32.1	0.0248	9.9	1.887
20	XYZ U2 U3 (S1,C2,C4,C6) M D Q O H HM HD k nosymm @N(2), nosymm @C1,C5, nosymm @C1,C5	276	7844	28.4	0.0244	8.6	1.858
21	XYZ U2 U3 (S1,C2,C4,C6) M D Q O H HM HD k nosymm @N(2), nosymm @C2, nosymm @C1,C5, nocon @tBu	401	7844	19.6	0.0234	5.3	1.739
22	XYZ U2 U3 (S1,C2,C4,C6) M D Q O H HM HD k nosymm @N(2), nosymm @C2, nosymm @C1,C5, nocon @all	435	7844	18	0.023	4.8	1.714

Table S5. Final Refinement.

Step	Parameter	#p	#d	D2p	$R1(F^2)$	Low : MP	GOF
**1-16**	As before						
**17**	**k'**	6	7844	1307	0.0250	0	1.869
**18**	XYZ U2 U3 (S1,C2,C4,C6) M D Q O H HM HD k nosymm @N(2) 3z@S1, nocon C1,C5	222	7844	35.3	0.0248	12.2	1.883

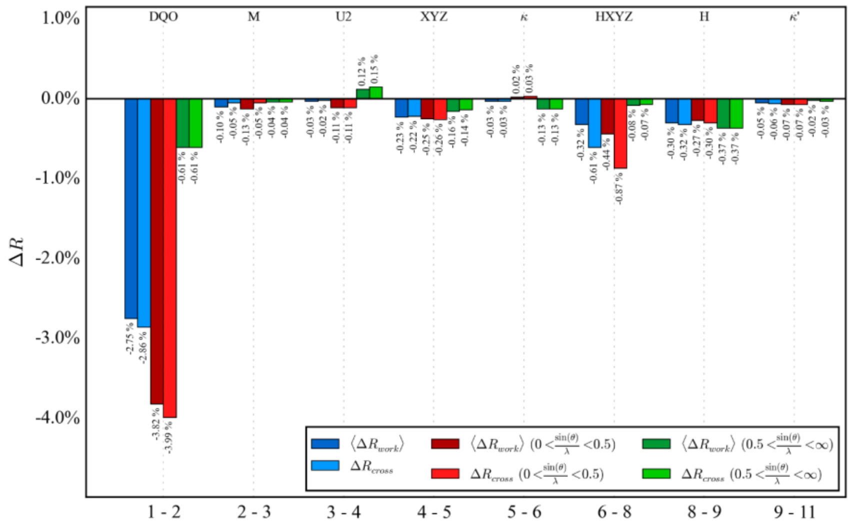

Figure S2. Rcross-Plot. Step 1-10.

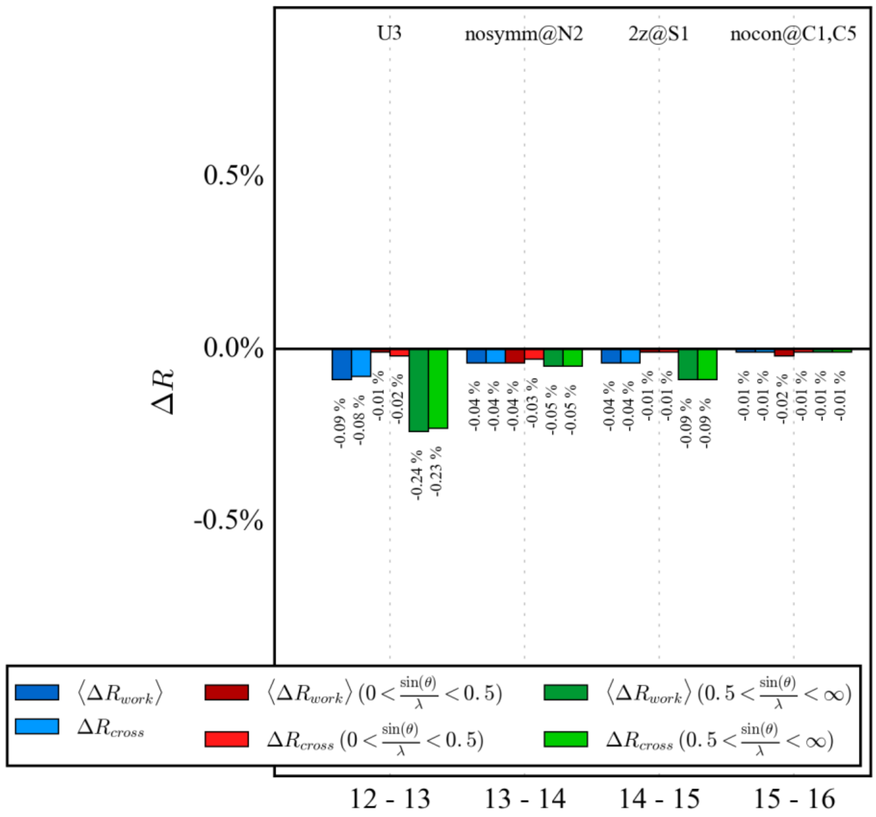

Figure S3 Rcross-Plot. Step 12-16.

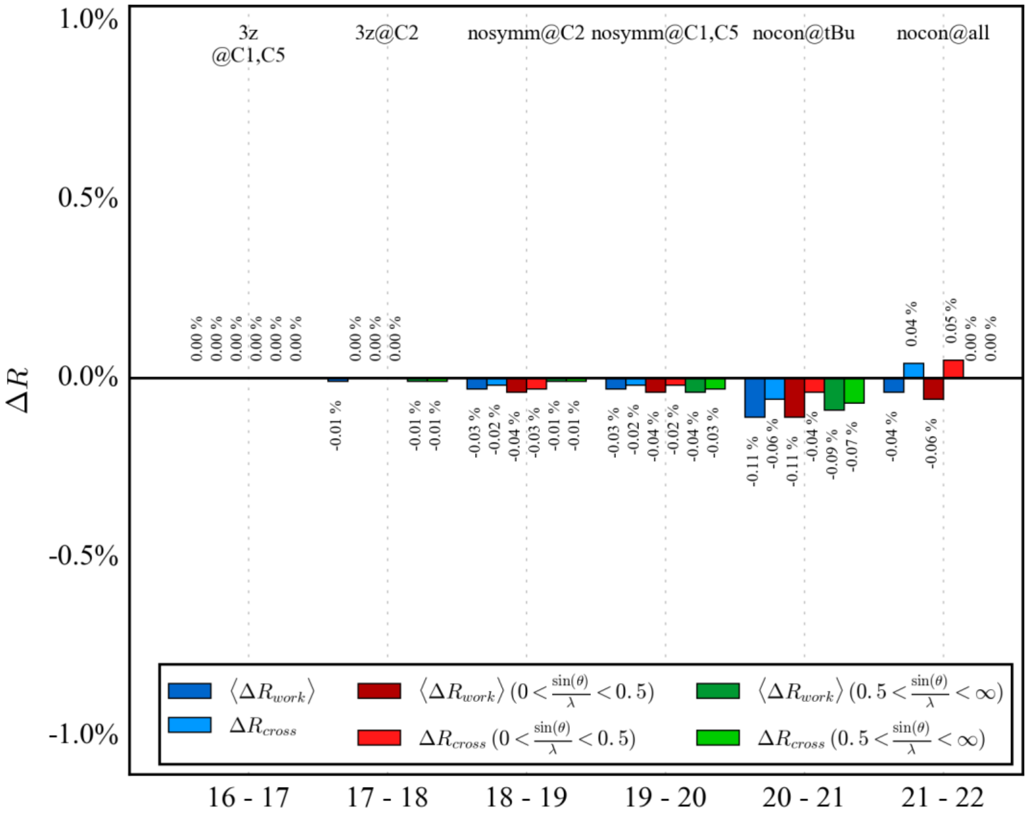

Figure S4 Rcross-Plot. Step 16-22.

S1-4.Refinement of anharmonic motion[13]

Table S6 The table below shows the minimum data resolution required for meaningful refinement of anharmonic thermal parameters (Gram-Charlier coefficients), for each atom refined with Gram Charlier coefficient.[14]

Atom[a]	Principal M.D.A's (A)			Min. resolution Qn	
S1	0.167	0.122	0.097	1.03	1.19
C2	0.222	0.175	0.111	0.79	0.92
C4	0.208	0.151	0.126	0.82	0.94
C6	0.247	0.149	0.118	0.79	0.92

Table S7 Calculated vibrational probability density function.

Atom	Total integrated negative probability [%]	Total integrated positive probability[%]	Maximum PDF value	Minimum PDF value	Integrated volume for negative probability [Å^3]	Integrated volume for positive probability [Å^3]
C(2)	-0.026	100.011	26382.27	-18.88	0.823	3.053
S(1)	-0.001	100.001	57010.51	-0.58	0.923	2.953
C(4)	-0.002	99.994	28430.52	-2.74	0.578	3.298
C(6)	0	99.976	26105.44	-0.78	0.626	3.250

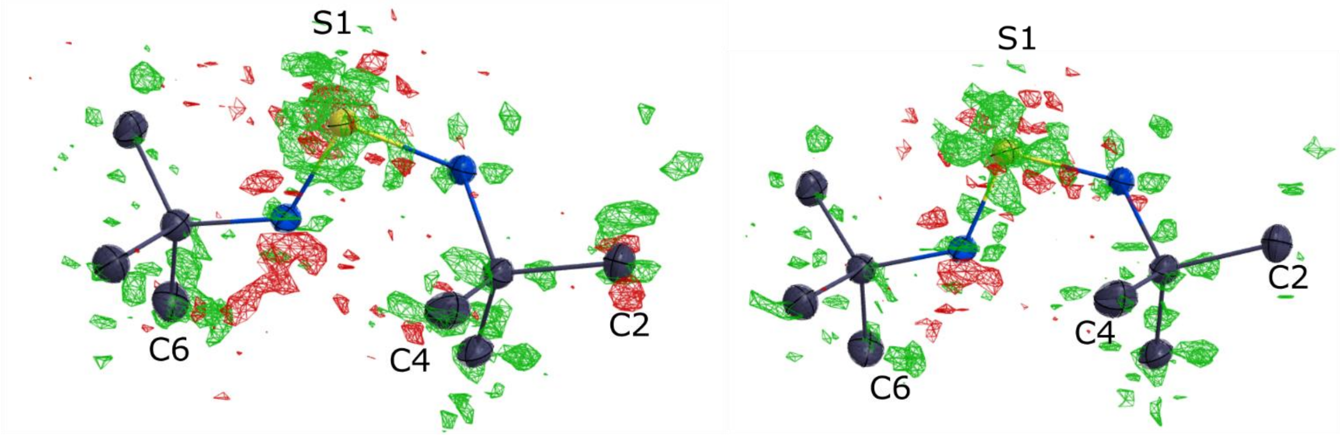

Figure S5 Residual density. The green density is positive and red negative (contour value ±0.074 e Å^{-3}) before (left) and after (right) anharmonic refinement with 3rd Gram-Charlier coefficients.

Table S8 Hirshfeld-test after final refinement step. Differences of the Mean-Square Displacement Amplitudes (DMSDA) (1 × 10-4 Å2) along atomic vectors.

Atom ->	Atom	Dist	DMSDA
S1	N1	1.6482	-2
N1	C1	1.4902	2
N2	C5	1.4776	8
C1	C2	1.5314	0
C5	C6	1.5308	1
S1	N2	1.5272	2
C1	C3	1.5291	-2
C1	C4	1.5295	1
C5	C7	1.5341	-2
C5	C8	1.5328	3

S1-5. Data quality [15]

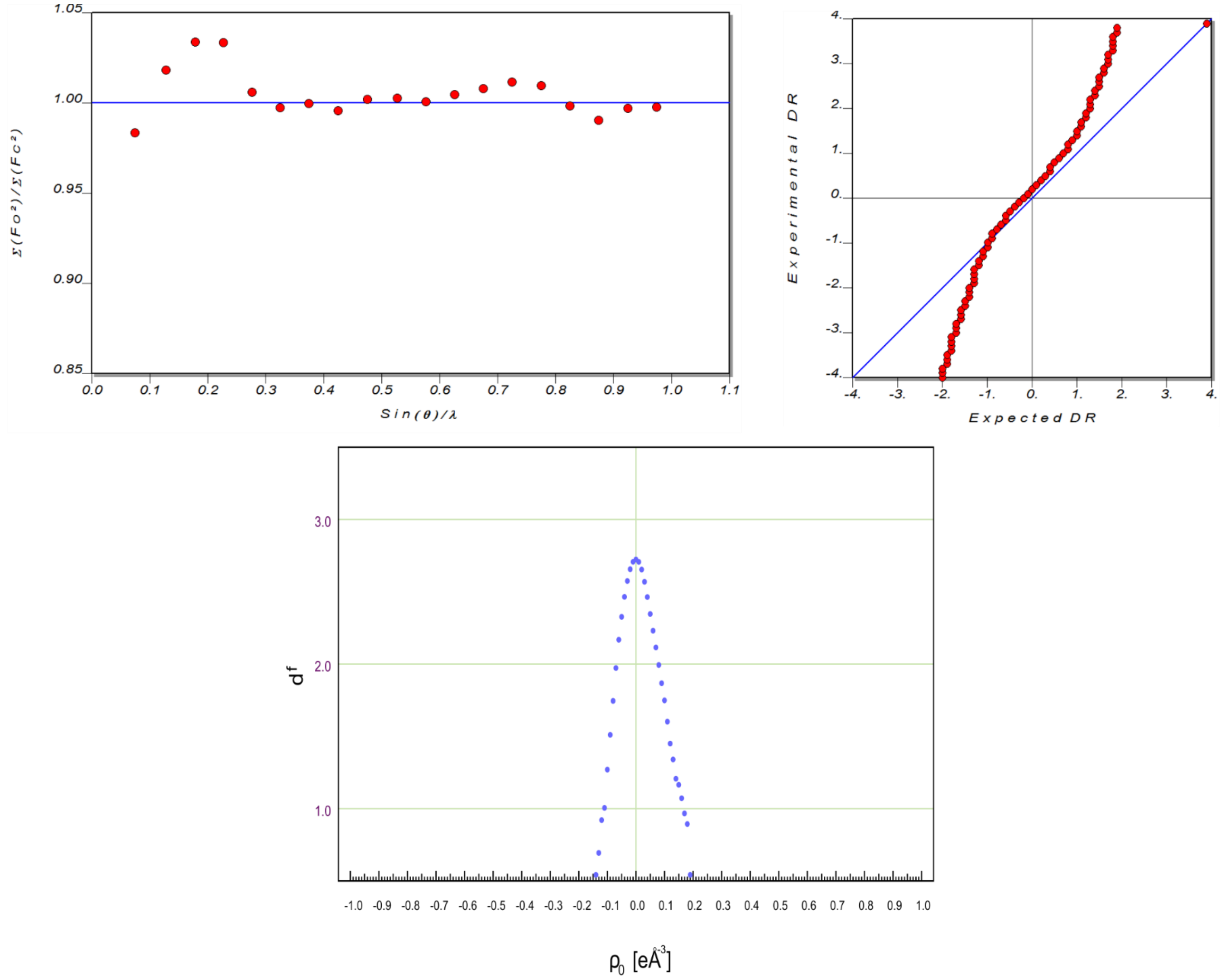

Figure S6 Drk-Plot, Normal Probability -Plot, Henn-Meindl-Plot, e_{gross}: 19.8 e

S1-6. Topological Analysis

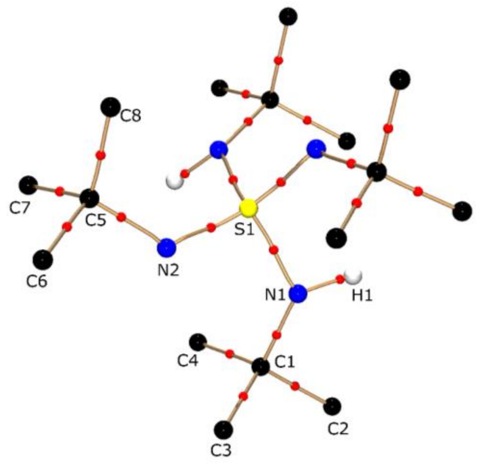

Figure S7. Molecular graph of **1**. Red: bond critical point.

Table S9 Topological parameters of selected bonds. Path length (R_{ij}), path length from atom to BCP (d1, d2), electron density ($\rho(r)_{BCP}$)), Laplacian ($\nabla^2\rho(r)_{BCP}$)) and ellipticity (ell) values at BCP. Estimated standard deviations were determined by the standard deviation of 20 cross-validation sets.[12]

Bond A1-A2	$\rho(r)_{BCP}$	$\nabla^2\rho(r)_{BCP}$	R_{ij}	d1	d2	ell
C1-C2	1.655(3)	-13.79(11)	1.5315(3)	0.7281(19)	0.803(2)	0.020(5)
C1-C3	1.658(3)	-13.99(11)	1.5291(2)	0.726(2)	0.804(2)	0.030(5)
C1-C4	1.650(3)	-13.80(11)	1.5299(4)	0.726(2)	0.804(2)	0.030(4)
C5-C6	1.645(3)	-13.24(12)	1.5310(3)	0.728(2)	0.803(2)	0.020(4)
C5-C7	1.636(3)	-13.03(12)	1.53420(18)	0.730(2)	0.804(2)	0.030(6)
C5-C8	1.634(3)	-12.92(12)	1.53310(17)	0.729(2)	0.804(2)	0.020(5)
N1-C1	1.642(7)	-9.00(17)	1.49030(13)	0.839(2)	0.651(2)	0.040(6)
N2-C5	1.626(6)	-10.4(2)	1.47810(12)	0.877(2)	0.601(2)	0.050(5)
S1-N1	1.808(6)	-14.8(3)	1.64860(15)	0.805(3)	0.843(2)	0.430(11)
S1-N2	2.173(4)	-21.2(3)	1.5291(2)	0.740(2)	0.789(3)	0.320(8)

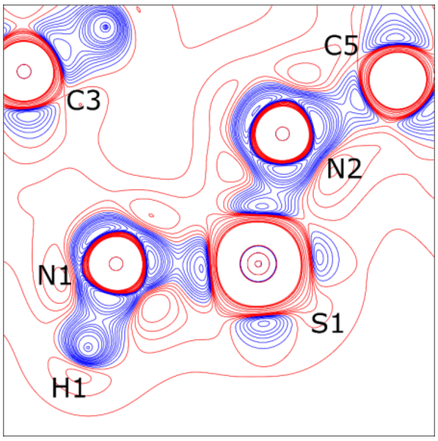

Figure S8 $\nabla^2\rho(r)$ distribution N1S1N2 plane. Contours are drawn at ± (1,2,5,7,10,15,30,50,70,100,115,135) $eÅ^{-5}$; blue contours show negative values, red contours show positive values.

Table S10 Maxima in $-\nabla^2\rho(\mathbf{r})$ around N1 and N2.

	Atom	$\nabla^2\rho(r)_{max}$ / $eÅ^{-5}$	$\rho(r)_{max}$ / $eÅ^{-3}$	r / Å
LP1	N2	-57.33	3.2886	0.4077
LP2	N2	-47.87	3.0965	0.4106
LP3	N1	-57.41	3.3471	0.4048

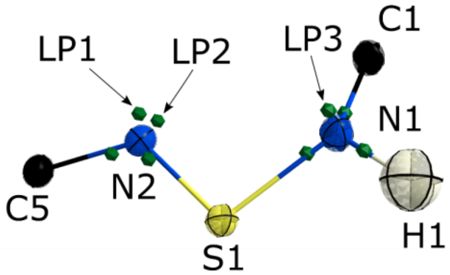

Figure S9 Maxima in Maxima in $-\nabla^2\rho(\mathbf{r})$ around N1 and N2.

S2. **NMR spectra**

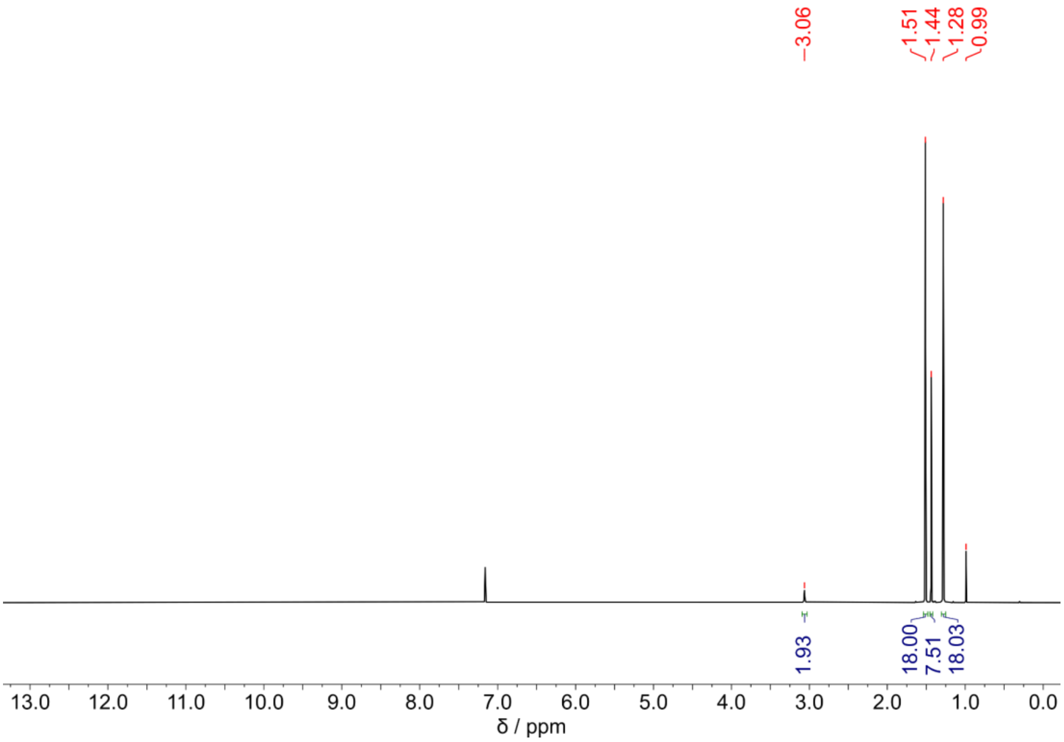

Figure S11 ^{1}H-NMR of **1** at 283 K in C_6D_6.

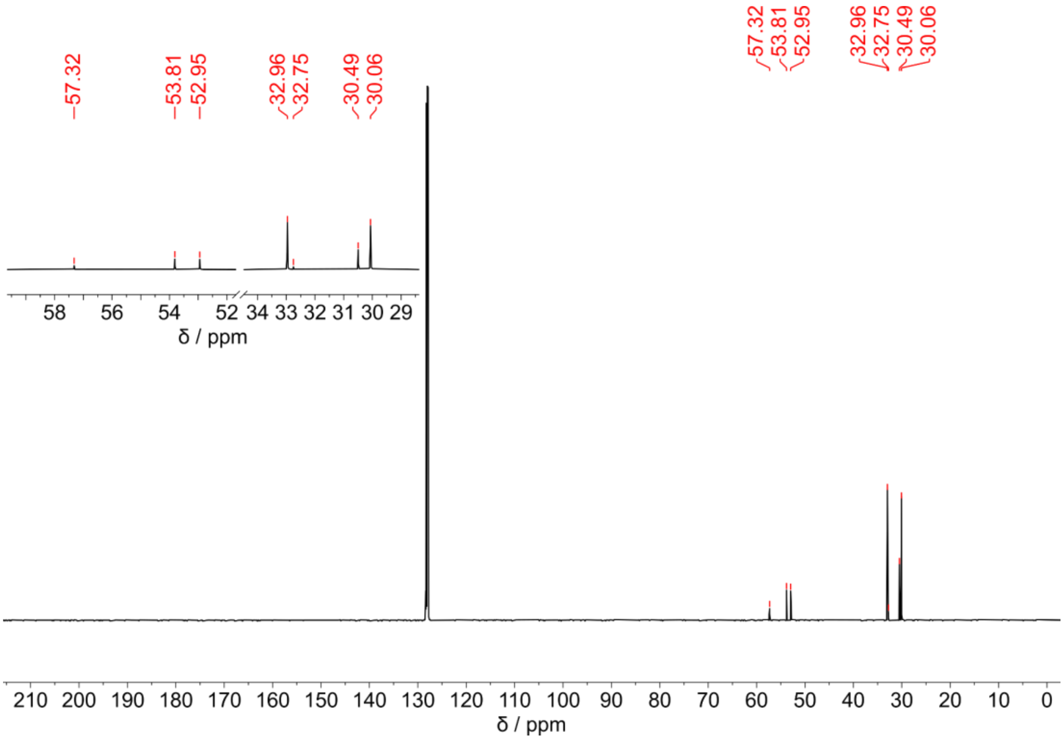

Figure S12 ^{13}C{^{1}H}-NMR of **1** at 283 K in C_6D_6.

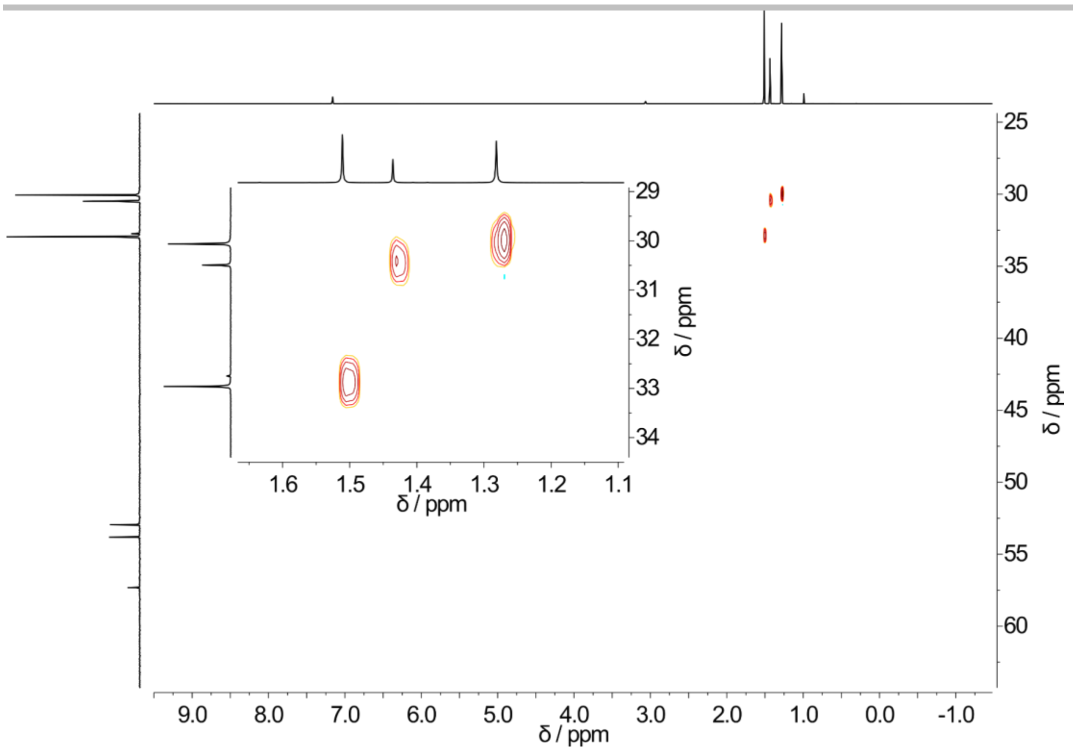

Figure S13 ^{13}C-^{1}H-HSQC-NMR of **1** at 283 K in C_6D_6.

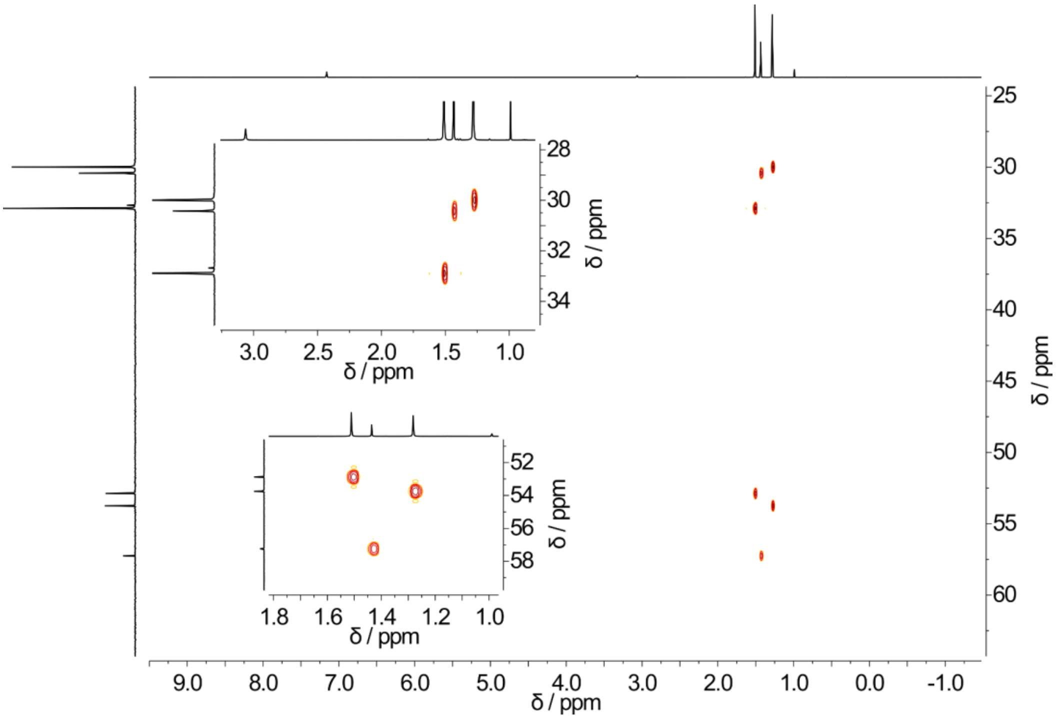

Figure S14 ^{13}C-^{1}H-HMBC-NMR of **1** at 283 K in C_6D_6.

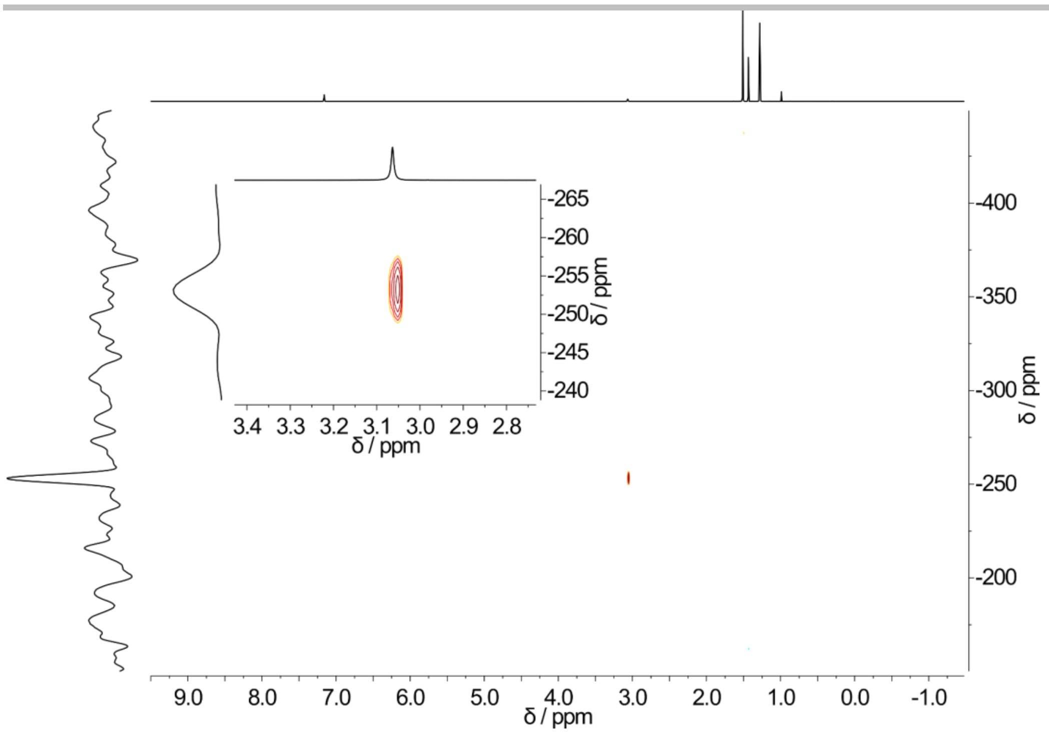

Figure S15 ^{15}N-^{1}H-HSQC-NMR of **1** at 283 K in C_6D_6.

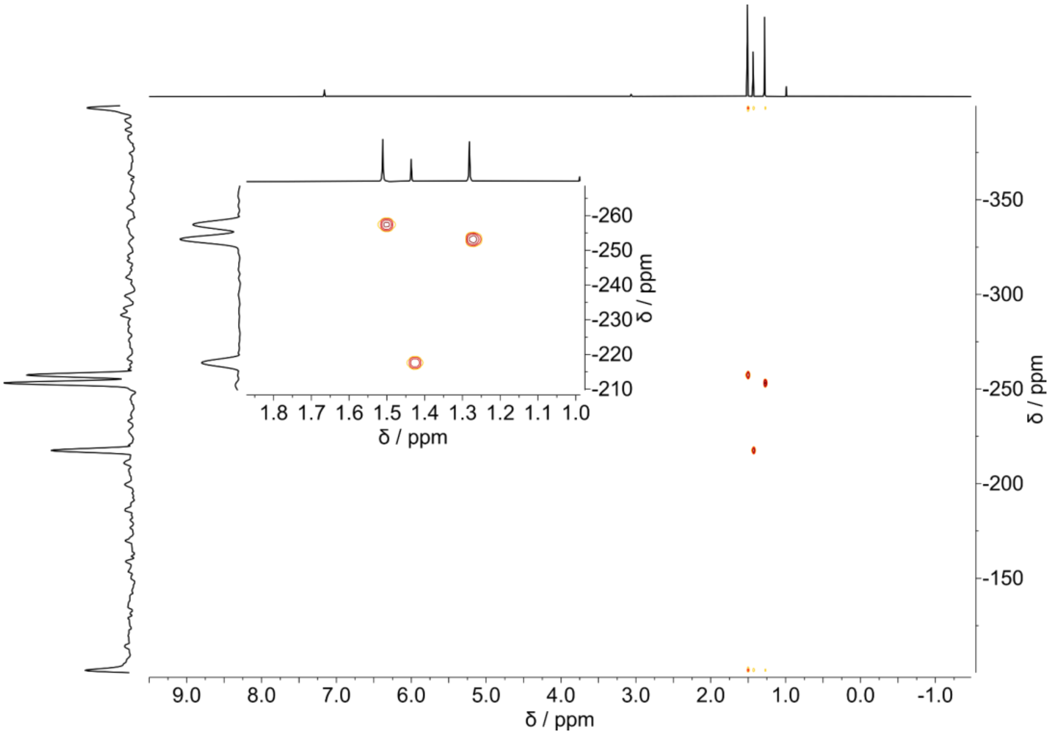

Figure S16 ^{15}N-^{1}H-HMBC-NMR of **1** at 283 K in C_6D_6.

S3. **Synthesis and decomposition of 1**

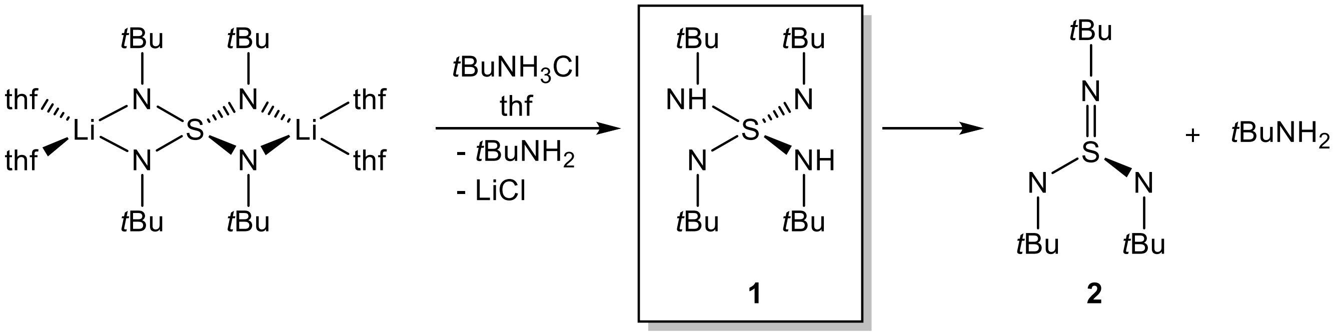

Scheme S1 Synthesis of the tetraimido sulfuric acid **1** from the lithiated precursor [(thf)$_4$Li$_2$(NtBu)$_4$S] via protonation with *t*BuNH$_3$Cl and the decomposition of **1** into *t*BuNH$_2$ and **2**.

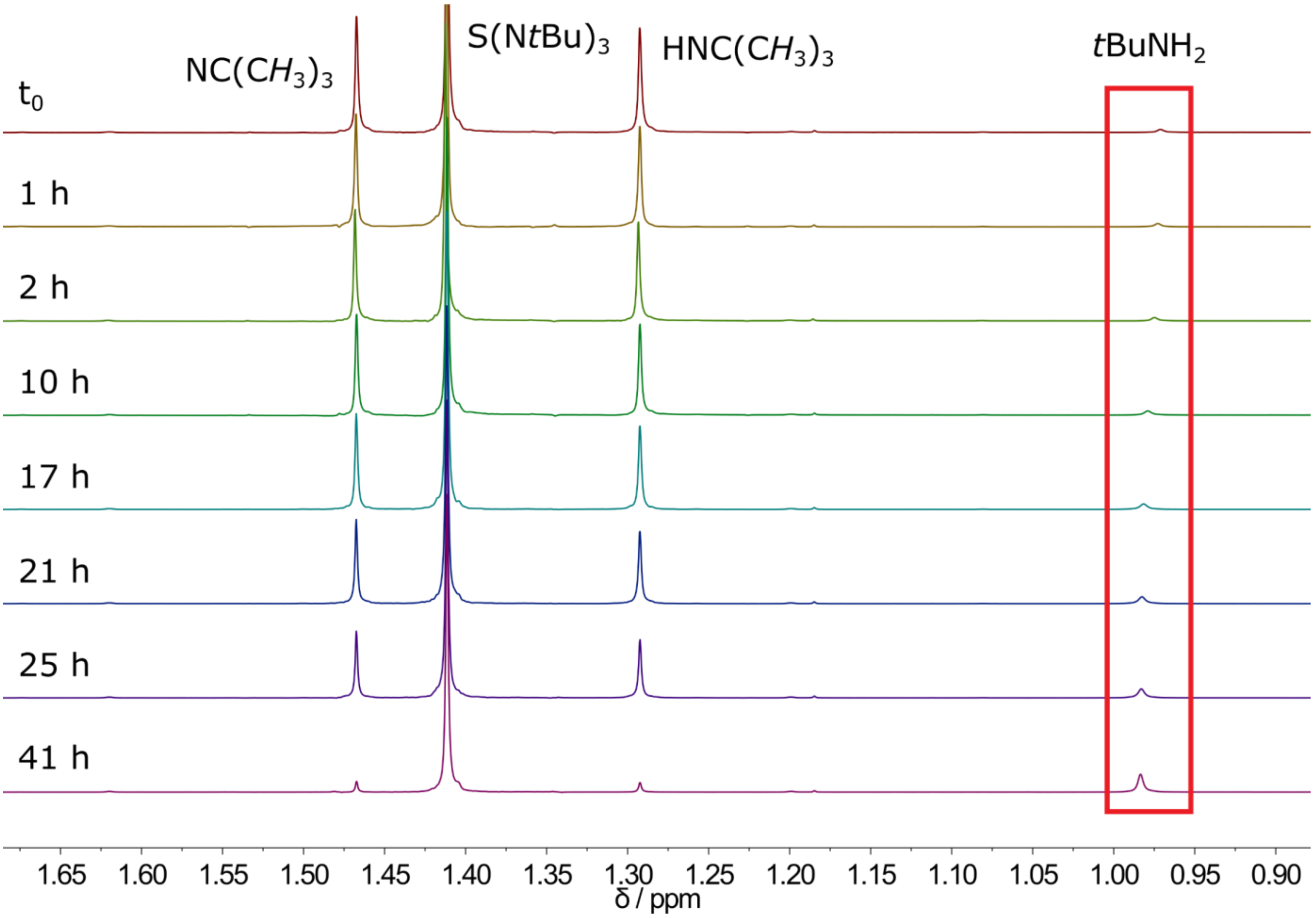

Figure S17 Stacked ^{1}H-NMR spectra of **1** at 283 K in toluene-d^8. Shown are the two signals of the *t*Bu groups of **1** and the two decomposition products S(N*t*Bu)$_3$ (**2**) and *t*BuNH$_2$ that increase over time.

[1] T. Kottke, D. Stalke, *J. Appl. Cryst.* **1993**, *26*, 615.
[2] Bruker AXS Inc., *SAINT*, Madison, **2016**.
[3] L. Krause, R. Herbst-Irmer, G. M. Sheldrick, D. Stalke, *J. Appl. Cryst.* **2015**, *48*, 3.
[4] L. Krause, R. Herbst-Irmer, D. Stalke, *J. Appl. Cryst.* **2015**, *48*, 1907.
[5] G. M. Sheldrick, *Acta Crystallogr.* **2015**, *A71*, 3.
[6] G. M. Sheldrick, *Acta Crystallogr.* **2015**, *C71*, 3.
[7] C. B. Hübschle, G. M. Sheldrick, B. Dittrich, *J. Appl. Cryst.* **2011**, *44*, 1281.
[8] A. Volkov, P. Macchi, L. J. Farrugia, C. Gatti, P. R. Mallinson, T. Richter, T. Koritsanszky, *XD2006. XD2006*, **2006**.
[9] a) Z. Su, P. Coppens, *Acta Crystallogr. Sec. A* **1998**, *54*, 646; b) P. Macchi, P. Coppens, *Acta Crystallogr. Sec. A* **2001**, *57*, 656.
[10] F. H. Allen, I. J. Bruno, *Acta Crystallogr. Sec. B* **2010**, *B66*, 380.
[11] R. Herbst-Irmer, J. Henn, J. J. Holstein, C. B. Hübschle, B. Dittrich, D. Stern, D. Kratzert, D. Stalke, *J. Phys. Chem. A* **2013**, *117*, 633.
[12] L. Krause, B. Niepötter, C. J. Schürmann, D. Stalke, R. Herbst-Irmer, *IUCrJ* **2017**, *4*, 420.
[13] a) R. Herbst-Irmer, *Acta Crystallogr. Sec. A* **2013**, *69*, 188; b) R. Herbst-Irmer, J. Henn, K. Meindl, *Acta Crystallogr. Sec. A* **2010**, *66*, s283.
[14] W. F. Kuhs, *Acta Crystallogr. Sec. A* **1992**, *48*, 80.
[15] a) V. V. Zhurov, E. A. Zhurova, A. A. Pinkerton, *J. Appl. Cryst.* **2008**, *41*, 340; b) Adam Stash, *DRKplot*, Moscow, **2007**; c) S. C. Abrahams, J. L. Bernstein, E. T. Keve, *J. Appl. Cryst.* **1971**, *4*, 284; d) K. Meindl, J. Henn, *Acta Crystallogr. Sec. A* **2008**, *64*, 404.
